# Long-term treatment with the PARP inhibitor niraparib does not increase the mutation load in cell line models and tumour xenografts

**DOI:** 10.1038/s41416-018-0312-6

**Published:** 2018-11-14

**Authors:** Ádám Póti, Kinga Berta, Yonghong Xiao, Orsolya Pipek, Gregory T. Klus, Thomas Ried, István Csabai, Keith Wilcoxen, Keith Mikule, Zoltan Szallasi, Dávid Szüts

**Affiliations:** 1 Institute of Enzymology, Research Centre for Natural Sciences, Hungarian Academy of Sciences, Budapest, Hungary; 2 Tesaro, Waltham, MA USA; 3 Department of Physics of Complex Systems, Eötvös Loránd University, Budapest, Hungary; 4 Genetics Branch, Center for Cancer Research, National Cancer Institute, Bethesda, MD USA; 5 Computational Health Informatics Program (CHIP), Boston Children’s Hospital, Boston, MA USA; 6 Harvard Medical School, Boston, MA USA; 7 Danish Cancer Society Research Center, Copenhagen, Denmark; 8 MTA-SE-NAP, Brain Metastasis Research Group, 2nd Department of Pathology, Semmelweis University, Budapest, Hungary

## Abstract

**Background:**

Poly-ADP ribose polymerase (PARP) inhibitor-based cancer therapy selectively targets cells with deficient homologous recombination repair. Considering their long-term use in maintenance treatment, any potential mutagenic effect of PARP inhibitor treatment could accelerate the development of resistance or harm non-malignant somatic cells.

**Methods:**

We tested the mutagenicity of long-term treatment with the PARP inhibitor niraparib using whole-genome sequencing of cultured cell clones and whole-exome sequencing of patient-derived breast cancer xenografts.

**Results:**

We observed no significant increase in the number and alteration in the spectrum of base substitutions, short insertions and deletions and genomic rearrangements upon niraparib treatment of human DLD-1 colon adenocarcinoma cells, wild-type and *BRCA1* mutant chicken DT40 lymphoblastoma cells and BRCA1-defective SUM149PT breast carcinoma cells, except for a minor increase in specific deletion classes. We also did not detect any contribution of in vivo niraparib treatment to subclonal mutations arising in breast cancer-derived xenografts.

**Conclusions:**

The results suggest that long-term inhibition of DNA repair with PARP inhibitors has no or only limited mutagenic effect. Mutagenesis due to prolonged use of PARP inhibitors in cancer treatment is therefore not expected to contribute to the genetic evolution of resistance, generate significant immunogenic neoepitopes or induce secondary malignancies.

## Background

Cells with defective homologous recombination (HR) due to mutation of the *BRCA1* or *BRCA2* genes are hypersensitive to the inhibition of poly-ADP ribose polymerase-1 (PARP-1).^1,2^ This led to the clinical development of PARP inhibitors as the first class of cancer therapeutics targeted against a DNA repair process.^3^

At the time of writing, three PARP inhibitor compounds are approved for treating ovarian cancer. Olaparib was the first approved agent that received approval for the treatment of germline *BRCA*-mutated advanced ovarian cancer that has received three or more prior lines of chemotherapy. Rucaparib was approved for the treatment of deleterious *BRCA* mutation (germline and/or somatic)-associated advanced ovarian cancer previously treated with two or more chemotherapies. More recently, niraparib was approved for the maintenance treatment of adult patients with recurrent epithelial ovarian, fallopian tube or primary peritoneal cancer who are in complete or partial response to platinum-based chemotherapy, without a restriction on germline *BRCA* status. Further clinical trials are aimed at extending the use of these drugs to breast cancer, prostate cancer and pancreatic cancer, to test further PARP inhibitor compounds, and to define patient pools that benefit from PARP inhibitor treatment in the absence of *BRCA1/2* germline mutations.^4–6^ The use of PARP inhibitors in a maintenance or adjuvant setting mandates a careful assessment of any late-onset side effects.

The PARP-1 enzyme, the primary target of PARP inhibitors, is involved in the repair of DNA single-strand breaks (SSBs). PARP inhibitors prevent the repair of SSBs by trapping the inactivated enzyme onto DNA, creating a block to replication and promoting the collapse of replication forks at SSBs.^7^ A further DNA damage tolerance function is attributed to PARP-1 in the rescue of stalled replication forks,^8,9^ which may contribute to the mechanism of synthetic lethality with *BRCA1* and *BRCA2*. Niraparib inhibits both PARP-1 and PARP-2 with low nanomolar half-maximal inhibitory concentration (IC_50_) values, selectively kills BRCA1/2 mutant cancer cells and traps PARP-1 more efficiently than olaparib.^10,11^

A potentially harmful side effect of the inhibition of DNA repair as a therapeutic strategy is increased mutagenesis in the treated cells. The genotoxic or mutagenic effect of PARP inhibition has mainly been examined in *BRCA1/2*-deficient cells, with reports of increased chromosomal instability^2,12,13^ and increased mutagenesis in a hypoxanthine-guanine phosphoribosyltransferase (HPRT) reporter assay in *BRCA2* mutant Capan-1 cells.^12^ In HR-proficient cells, PARP inhibition increases sister chromatid exchange (SCE) formation and olaparib has also been shown to induce chromatid-type chromosome aberrations.^14,15^

In contrast with the above findings, preclinical toxicology results of clinically used PARP inhibitors reported no mutagenic effect in the bacterial Ames test, while carcinogenicity was not investigated. The Ames test is of limited relevance for this class of drugs, as prokaryotes do not have PARP enzymes involved in DNA repair. It was, therefore, important to obtain a comprehensive view of genomic changes elicited by PARP inhibitors. Whole-genome sequencing (WGS) of cultured cells following drug treatment offers a convenient method for this purpose, which we successfully used to determine and compare the mutagenic effect of several common anticancer cytotoxic agents, and demonstrate the mutagenicity of cisplatin.^16^ In this study, we subjected *BRCA* wild-type (WT) and *BRCA* mutant cell lines to long-term treatment with the PARP inhibitor niraparib. WGS analysis of post-treatment cell clones did not reveal increased mutagenesis, with subtle exceptions. The lack of mutagenic effect was confirmed in vivo using patient-derived breast cancer xenograft (PDX) tumours.

## Methods

### Cell culture

The following cell lines were used: WT and *BRCA1*^*−/−*^ DT40 as used previously,^17^ DLD-1 and 184B5 (ATCC) and SUM149PT (Asterand Bioscience). All cell lines were tested for mycoplasma contamination, and validated using the WGS data obtained during this work. DT40 cells were cultured in RPMI-1640 medium (Lonza) supplemented with 7% foetal bovine serum and 3% chicken serum; DLD-1 and 184B5 cells were cultured in RPMI-1640 medium supplemented with 10% foetal bovine serum; and SUM149PT cells were cultured in Ham’s F12 medium (Sigma) supplemented with 5% foetal bovine serum, 10 mM HEPES-NaOH (pH 7.4), 1 μg/ml hydrocortisone and 5 μg/ml insulin (all from Sigma). All cells were grown at 37 °C under 5% CO_2_.

### PARP inhibitor treatments

Niraparib was obtained from Tesaro and dissolved in dimethyl sulfoxide (DMSO) at 10 mM. Cytotoxicity assays were performed in 96-well cell culture plates. DT40 cells were plated at 5000 cells/well, and niraparib was added at the time of plating. Measurements were taken after 3 days on a Perkin-Elmer EnSpire instrument, 2 h following the addition of 5% PrestoBlue reagent (Thermo Fischer Scientific) to the medium. DLD-1 and SUM149PT cells were plated at 1000 and 3000 cells/well, respectively. The treatment was started one day after plating (day 0), and the medium was changed on days 3 and 6 with the inclusion of fresh niraparib. Measurements were taken on day 8 as above, except that the growth medium was replaced with phosphate-buffered saline containing 5% PrestoBlue. The data were evaluated using GraphPad Prism.

Growth rates were calculated from daily cell counts. Long-term niraparib treatments were performed over 30 days in 24-well cell culture plates. Growth medium with or without freshly diluted niraparib was replaced three times per week, and cells were passaged two or three times per week as necessary. Cisplatin was used at 10 μM for 1 h once a week for four cycles as described.^16^ Cells were cloned by limiting dilution. Genomic DNA preparations were made from one to two million cells using the Gentra Puregene method (Qiagen).

### SCE assay

Niraparib and olaparib (Selleckchem) were added from 1 mM stock solutions in DMSO to produce a final concentration of 500 nM and a final DMSO concentration of 0.05%. SCE assays of DLD-1 and 184B5 cells exposed to the various treatments were performed essentially as described,^18^ with a 5-bromo-2′-deoxyuridine (BrdU) exposure duration of 43 and 40 h, respectively, and with the BrdU exposure occurring at the same time as the treatments. Modifications to the above protocol were (i) the use of colcemid at 0.05 µg/ml for 90 min rather than at 0.02 µg/ml for 4 h; (ii) the preparation of metaphase spreads by the method described by Padilla-Nash et al.,^19^ with the spreading of the cells on slides performed in a Cytogenetic Drying Chamber (Thermotron, Holland, MI 49423 USA) at approximately 23 °C and with the relative humidity of the chamber set at 47%.

### PDX treatment

Outbred athymic (nu/nu) female mice (Hsd:Athymic Nude-Foxn1^nu^) weighing 18–25 g (Harlan Laboratories, Gannat, France) were subcutaneously implanted with HBCx xenografts. When tumours reached a size of 70–200 mm^3^, mice were assigned to homogeneous groups of five animals and were dosed by oral gavage daily at 50 mg/kg. A compound was prepared at least 48 h before administration by dissolution of powder by constant stirring (and sonication when it was necessary) in 0.5% methylcellulose at 10 mg/ml. Tumours were collected 4 h after the last dosing on day 28. Tumour volume was evaluated by measuring biweekly tumour diameters with a caliper. The formula tumour volume = (length x width^2^)/2 was used, where the length and the width were the longest and the shortest diameters of each tumour, respectively. Animals were euthanised if the tumour volume exceeded 2000 mm^3^. Extracted tumour samples were formalin-fixed and paraffin-embedded according to standard methods.

### DNA sequencing, mutation calling and data analysis

WGS was done at Novogene (Beijing, China). PDX sequencing was done at BGI Americas (Cambridge, MA, USA). The alignment of reads was done as described.^17^ The PDX-derived human and mouse sequences were separated during the alignment process.

Independently arising SNVs and short indels were identified using IsoMut,^20^ using default settings adjusted for copy number for WGS, and the criteria of minimum three supporting reads and exonic location for PDX mutations. Short deletions were classified as repeat if the deleted sequence was present in at least two tandem copies, and as microhomology if the sequence at the two breakpoints contained at least one base pair of homology. Structural variations were detected using CREST^21^ with post-filtering steps (Supplementary Methods).

Two-sided unpaired *t* tests were used for statistical comparisons of mutation numbers with no adjustments for multiple comparisons.

## Results

### Modelling long-term PARP inhibitor treatment in cell lines

**Fig. 1:**
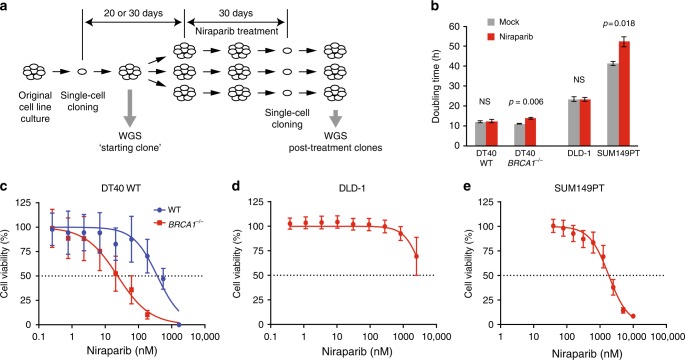
Long-term niraparib treatments. **a** A schematic outline of the long-term treatment experiments. Single-cell clones were expanded over a period of 20 days (DT40 cell lines) or 30 days (DLD-1 and SUM149PT cell lines). A sample was taken for sequencing as soon as a sufficient number of cells was available (“starting clone”). Further single-cell cloning was performed at the end of the treatment; one clone was sequenced from each treated cell population. **b** Doubling time of the indicated cell lines during continuous mock or niraparib treatment. The *p* values of significant changes are shown (*t* test). **b** A comparison of the niraparib sensitivity of WT and *BRCA1*^*–/–*^ DT40 cell lines using cytotoxicity assays. **c**–**e** Measurement of the niraparib sensitivity of the experimental cell lines using cytotoxicity assays. The mean and SEM of three independent experiments is shown in **b**–**e**. NS not significant

We modelled preclinical and clinical in vivo niraparib treatments in cell lines by continuous exposure to niraparib for 30 days. To avoid analysing non-independent cells derived from potential drug-resistant subclones that may emerge during the treatment, we isolated only a single-cell clone from each population for analysis (Fig. 1a).

Four cell lines were selected for this study. Our earlier demonstration of an in vitro reproduction of spontaneous and *BRCA* defect-associated mutagenic processes observed in human tumours^17^ provided the rationale to use an isogenic pair of WT and *BRCA1*^*−/−*^ mutant DT40 chicken lymphoblastoma cell lines for the specific investigation of the effect of *BRCA1* loss. A further benefit of using WT DT40 cells is their low spontaneous mutation rate,^16^ which makes them a good model for mutagenesis in somatic cells. We selected the DLD-1 human colorectal carcinoma cell line for its normal karyotype and a high mutagenic rate due to microsatellite instability,^22^ and the SUM149PT *BRCA1* mutant triple-negative breast carcinoma cell line for highest relevance to PARPI treatment and no detectable BRCA1 protein.^23,24^

Long-term niraparib treatment concentrations were established using cytotoxicity assays. Niraparib selectively killed the *BRCA1*^*–/–*^ DT40 cells, with IC_50_ concentrations for the WT and the *BRCA1*^*–/–*^ mutant measured as 369 nM (95% confidence interval (CI): 154–796 nM) and 24 nM (95% CI: 11–51 nM), respectively (Fig. 1c). DLD-1 was insensitive to niraparib treatment, with an IC_50_ in excess of 4000 nM (Fig. 1d), and the *BRCA1* mutant SUM149PT also showed fairly low sensitivity despite the presence of the 2288delT frameshift mutation, with an IC_50_ of 1841 nM (95% CI: 1426–2366 nM, Fig. 1e). We chose a treatment concentration of 500 nM for the treatment of WT DT40, DLD-1 and SUM149PT cells, which is around the peak plasma concentration measured in patients receiving a daily oral dose of 300 mg,^25^ and 50 nM for the DT40 *BRCA1*^*–/–*^ cells. The treatments slightly slowed the growth of the SUM149PT and DT40 *BRCA1*^*–/–*^ lines (Fig. 1b), and appeared to lead to reduced niraparib sensitivity in SUM149PT post-treatment cell clones, but not in the other cell lines (Supplementary Fig. S1). No significant cell death was observed during the treatment, suggesting that limited selection was involved in the isolation of post-treatment clones.

### Single-nucleotide variations in cell lines

Treatment-induced mutations were identified using the IsoMut tool^20^ using separately optimised mutation filters for genomic regions with distinct ploidy levels. We found that DT40 and DLD-1 were largely diploid, whereas the SUM149PT cell line was aneuploid, and most of the diploid regions showed loss of heterozygosity (Supplementary Fig. S2).

**Fig. 2:**
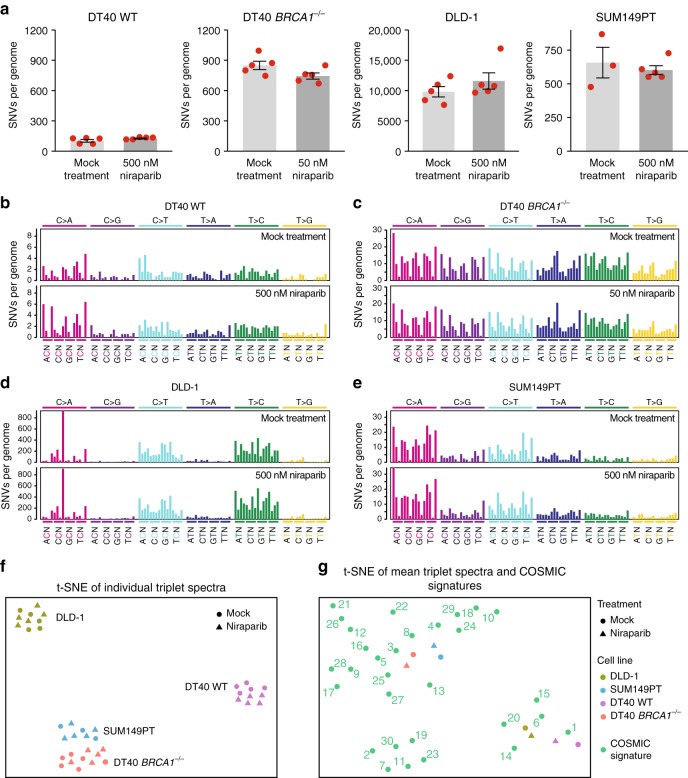
SNVs generated during long-term niraparib treatment. **a** The mean number of SNVs generated per sequenced genome in each indicated cell line following mock treatment or treatment with the indicated concentration of niraparib. Red symbols show the values for individual samples, error bars indicate SEM. **b**–**e** Mean triplet SNV mutation spectrum of the mock treatment (top panel) or niraparib treatment (bottom panel) in the indicated cell lines. Each mutation class, as indicated at the top of the panel, is separated into 16 categories based on the identity of the preceding and following nucleotide as shown below. The order of the following nucleotides, not shown due to lack of space, is alphabetical. **f** Similarities and differences of the triplet SNV spectra of individual samples visualised using *t*-distributed stochastic neighbour embedding (*t*-SNE). **g** Comparison of the mean triplet SNV spectra shown in **b**–**e** to COSMIC mutation signatures using *t*-SNE. Experimental spectra are labelled according to the key shown on the right; COSMIC signatures are numbered

Any difference in SNVs between the mock-treated and niraparib-treated clones should show the mutagenic effect of the drug. We identified 102 ± 29 (SD) spontaneous base substitutions in mock-treated WT DT40 cells, not significantly different from 125 ± 11 SNVs found after niraparib treatment (Fig. 2a and Table S1, *p* = 0.15, *t* test). The number of spontaneous SNVs was about eight-fold higher in *BRCA1*^*−/−*^ mutant cells (849 ± 93) in agreement with our earlier results,^17^ and again similar following niraparib treatment (Fig. 2a, b). The non-significant 12% decrease in the mean number of SNVs to 744 ± 31 (*p* = 0.077) may be connected to the slower growth of the niraparib-treated cell pools (Fig. 1b). DLD-1 cells showed a high level of spontaneous SNV mutagenesis with 9799 ± 1910 genomic mutations acquired over a 60-day culture period, which did not significantly change upon niraparib treatment (Fig. 2a and Table S1, *p* = 0.29). The *BRCA1* mutant SUM149PT breast cancer cells had a lower mutation rate with 608 ± 146 acquired base substitutions, which again did not alter due to niraparib treatment (Fig. 2a and Table S1, *p* = 0.56). In contrast, weekly repeated treatments with 10 μM cisplatin were strongly mutagenic on WT DT40 cells (Supplementary Fig. S3), in agreement with our earlier results,^16^ and the same treatment regimen also resulted in a significant increase of SNVs in the case of *BRCA1*^*−/−*^ mutant cells (Supplementary Fig. S3, *p* = 0.034, *t* test).

The triplet SNV spectra, showing each base substitution in the context of the neighbouring bases, also did not reveal any mutagenic effect of niraparib on either WT or *BRCA1*^*−/−*^ mutant DT40 cells (Fig. 2b–e and Supplementary Fig. S4). A visualisation of the similarities of individual triplet spectra using *t*-distributed stochastic neighbour embedding (*t*-SNE) clearly separates spectra from different cell lines, but clusters the mock-treated and niraparib-treated samples together, again indicating a lack of mutagenic effect (Fig. 2f).

Our study also provides the first characterisation of mutagenic processes in the DLD-1 and SUM149PT cell lines. The comparison of triplet SNV spectra with triplet mutation signatures derived from cancer genomes^26,27^ using *t*-SNE showed closest correlation of the WT DT40 spontaneous spectrum with the ageing-associated signature 1, whereas SNV mutagenesis in the DT40 *BRCA1*^*−/−*^ cells was best correlated with signature 3 typical of BRCA1/2 mutant cancers (Fig. 2g) as published earlier.^16,17^ The calculation of Pearson correlations or hierarchical clustering supports these findings (Supplementary Fig. S5). In DLD-1 cells, the pattern of spontaneous mutagenesis best correlated with signature 6, followed by signatures 20 and 15 (Fig. 2g). These signatures were found to associate with defective DNA mismatch repair (MMR),^26^ and the correlation is explained by the presence of frameshift mutations in each allele of the key MMR gene *MSH6* in the DLD-1 genome.^22^ Spontaneous SNV mutagenesis in the SUM149PT cell line showed best correlation with signatures 4 and 8 (Fig. 2g and Fig. S4). Unlike in the DT40 *BRCA1*^*−/−*^ cells, mutagenesis in the *BRCA1* mutant SUM149PT cells showed only weak or no correlation with the *BRCA* defect-associated signature 3. Therefore, despite the presence of the homozygous 2288delT *BRCA1* mutation, SUM149PT cells do not have a *BRCA1*-deficient SNV mutagenesis phenotype. Together with the low sensitivity to niraparib, this suggests that suppressor mutations may have arisen in the tumour or the cell line, but an analysis of the coding mutations did not reveal any alterations in known HR-interacting genes to explain the limited *BRCA1*-like phenotype of SUM149PT.

### Indels and large rearrangements in cell lines

**Fig. 3:**
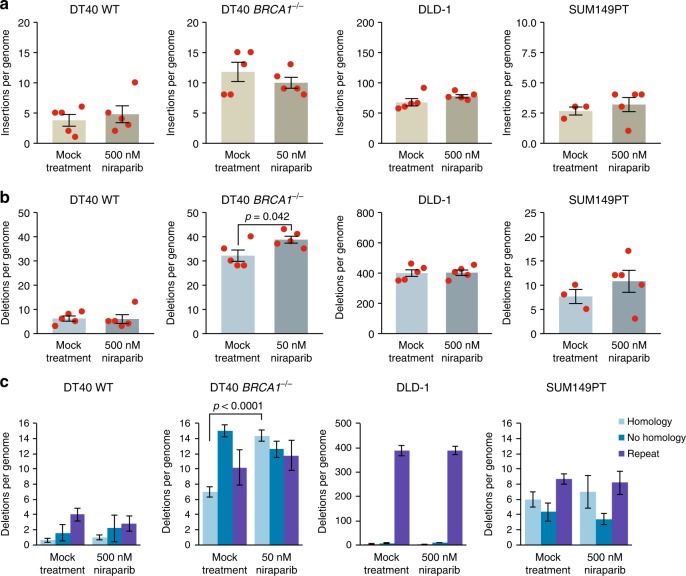
Indels generated during long-term niraparib treatment. **a**, **b** The mean number of short insertions (**a**) or short deletions (**b**) generated per sequenced genome in each indicated cell line following mock treatment or treatment with the indicated concentration of niraparib. Red symbols show the values for individual samples. **c** A classification of detected short deletion events by sequence context. The minimum length of classified microhomologies was 1 bp. Error bars indicate SEM in all panels. Significant differences are indicated (*p* < 0.05, unpaired *t* test)

We catalogued all short indels up to 50 bp, and found no significant difference in the number of insertions or deletions between the mock-treated and niraparib-treated samples in the investigated cell lines apart from a small but significant increase in the number of deletions in DT40 *BRCA1*^*−/−*^ (Fig. 3a, b and Table S1). We classified the deletions according to sequence context. The high level of short deletions at repeat sequences in the DLD-1 cell line confirmed its microsatellite instability phenotype (Fig. 3c). In the *BRCA1*^*−/−*^ mutant DT40 cells, we found more deletions of each category than in the WT, in agreement with earlier results.^17^ In the *BRCA1*^*−/−*^ cells we found a significant increase in events with one or more base pairs of microhomology between the ends of the deletion upon niraparib treatment (*p* < 0.0001, *t* test), though there was no similar effect in the other cell lines (Fig. 3c and Table S1). The increase in microhomology-mediated deletions was specific to the *BRCA1* mutant DT40 cells, which might be due to an increased use of nonhomologous end joining in the absence of HR at double-strand breaks resulting from the collision of replication forks with trapped PARP enzymes.

**Fig. 4:**
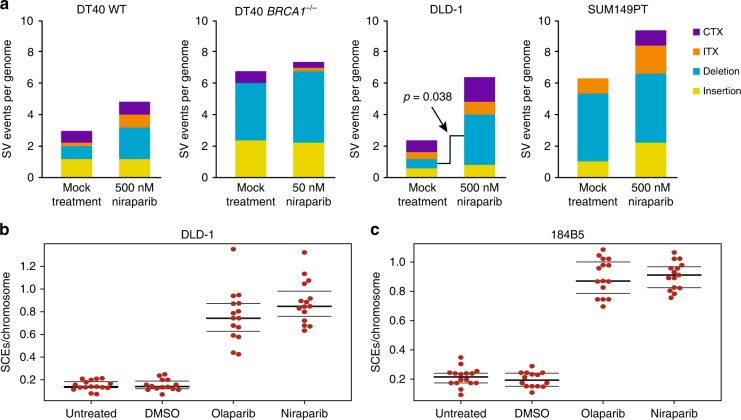
Large rearrangements and sister chromatid exchanges generated during long-term niraparib treatment. **a** The mean number of structural variations (SV) per genome derived from CREST analysis, separately showing large insertions, large deletions, intrachromosomal rearrangements (ITX) and interchromosomal rearrangements (CTX). Error bars indicate SEM in all panels. Significant differences are indicated (*p* < 0.05, unpaired *t* test), all other pairwise comparisons between the mock-treated and niraparib-treated values were not significant. **b**, **c** The number of SCEs measured in DLD-1 cells (**b**) and 184B5 cells (**c**) following treatment with 500 nM niraparib or 500 nM olaparib is shown as SCEs per number of chromosomes in each cell. Black lines indicate the median and the lower and upper quartiles

There were few instances of larger insertions, deletions or chromosomal rearrangements in the sequenced genomes. In general, there was no significant difference between the control and the niraparib-treated samples, except for an increase in large deletions in DLD-1 samples upon niraparib treatment (*p* = 0.038, *t* test) (Fig. 4a).

SCEs are theoretically non-mutagenic rearrangements, and the documented elevation of SCE numbers upon PARP inhibition is likely due to increased HR providing a back-up for defective SSB repair. We tested whether the treatment conditions used in the in vitro experiments induce SCEs. Indeed, the treatment of either DLD-1 cells or the 184B5 chemically immortalised non-malignant breast epithelial cell line with 500 nM niraparib or olaparib induced approximately four times more SCEs than seen in the untreated controls (Fig. 4b, c). Contrasting the high the rate of SCE formation at about 40/cell cycle in niraparib-treated DLD-1 cells with the lack of observed SNV and indel mutagenesis over approximately 30 cell divisions indicates that the niraparib-induced formation of SCEs is essentially non-mutagenic.

### Niraparib treatment of breast cancer xenografts

**Fig. 5:**
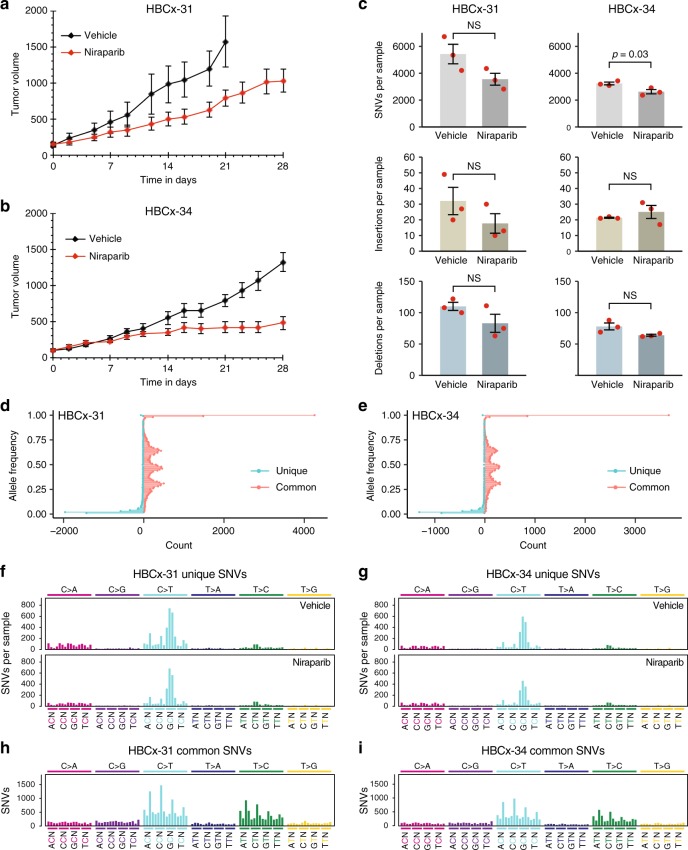
The effect of niraparib on the formation of subclonal tumour-specific mutations in PDX models. **a**, **b** Tumour growth in animals implanted with the indicated PDX model. The mean and SEM of four independent samples is shown. **c** The mean number of unique SNVs (top panel), short insertions (middle panel) and short deletions (bottom panel) identified in whole-exome sequence data from tumour samples derived from control or niraparib-treated animals. Red symbols show the values for individual samples; error bars indicate SEM. Significant differences are indicated (*p* < 0.05, unpaired *t* test); NS not significant. **d**, **e** Allele frequency distribution of SNVs unique to individual samples (blue, the mean of all six sequenced samples of each PDX is shown), and of SNVs common to all samples of the respective PDX (red). **f**, **g** Mean triplet mutation spectrum of unique SNVs in the indicated vehicle-treated (top panel) or niraparib-treated (bottom panel) PDX samples. **h**, **i** Triplet mutation spectrum of common SNVs in the indicated PDX samples. The labelling of panels **f**–**i** is as described under Fig. 2

To confirm the cell line-derived results in vivo, we subjected mice implanted with breast cancer-derived xenografts to 28 days of niraparib treatment, and performed WGS on the extracted tumour. Two *BRCA1* WT PDX models were used, HBCx-31 derived from a triple-negative invasive ductal carcinoma^28^ and the oestrogen-dependent HBCx-34.^29^ Niraparib treatment slightly slowed the growth of each PDX, but did not decrease their size (Fig. 5a, b). Using IsoMut with permissive settings, we looked for subclonal mutations unique to each tumour sample; as such mutations would be expected to arise during the treatment. After careful separation of human and mouse sequences, we identified unique SNVs and indels with low allele frequency in all samples (Fig. 5d, e).

There was no increase in the number of mutations or a change in mutation spectra when comparing control and niraparib-treated samples, indicating that niraparib treatment did not generate detectable subclonal mutations in vivo (Fig. 5c, f, g). Note that the spectrum of non-unique SNVs common to all samples of each PDX is substantially different from the spectrum of unique mutations (Fig. 5f–i), suggesting that the detection of unique subclonal mutations was not contaminated by variations present in the germline or the original tumour. The unique subclonal mutations, therefore, indeed reflect ongoing mutagenesis specific to the tumour, and could have reasonably been expected to show any potential mutagenic effect of niraparib. The approach of using high coverage WGS exome sequencing, necessary for the detection of low allele frequency subclonal mutations, precludes the analysis of subclonal structural variations in the investigated tumour samples. The results from these *BRCA1* WT PDX models also serve to model mutagenesis in the somatic tissue of patients with *BRCA*-deficient tumours.

## Discussion

This work demonstrated that continuous treatment of various cell lines and tumour xenografts with the PARP inhibitor niraparib does not induce genomic SNV mutations, and also does not induce small indels in *BRCA*-proficient cells.

Genomic mutations can arise spontaneously during cell proliferation, or due to exogenous sources. Of the four investigated cell lines, WT DT40 has the lowest spontaneous mutation rate, similar to the spontaneous mutation rates reported in a number of organisms.^16^ The high sensitivity and specificity of the employed mutation detection methods were demonstrated earlier: with the IsoMut tool over 90% of SNVs and short indels can be detected with a near-zero false-positive rate when multiple whole genomes are analysed together.^20^ The assay was powered to detect an approximately 25% increase over this background level, but niraparib caused no significant increase, suggesting that clinical PARP inhibitor treatment could at most marginally increase spontaneous rates of mutagenesis. While the spontaneous mutation rates in the other cell lines were higher due to DNA repair defects, the results support the same conclusion.

The spontaneous mutations were acquired over a period encompassing over 100 cell divisions in DT40 cells, 50 cell division in DLD-1 cells and about 30 cell divisions in the slow growing SUM149PT cells. Somatic cells typically take years to go through this number of divisions,^30^ and the correlation of cancer risk with stem cell divisions suggests that mutations are mainly acquired during active cell cycles,^30^ probably during DNA replication. With the assumption that this would also apply to niraparib-induced mutations, our experiments may have modelled years of treatment. In contrast with the lack of mutagenesis upon niraparib treatment, the same cell culture-based assay demonstrated a high level of mutagenesis over the same period due to the alkylating agents cisplatin, cyclophosphamide and methyl methanesulfonate, and a low level following etoposide treatment.^16,17^ Moreover, our results showed an even stronger mutagenic effect for cisplatin in *BRCA1*-deficient cells. Currently, platinum agents precede PARP inhibitors in the treatment of ovarian cancer,^31^ and are also used to treat *BRCA* mutant triple-negative breast cancer. Replacing platinum by non-mutagenic alternatives such as PARP inhibitors will likely reduce the mutational load of tumour and normal cells and thus reduce both the level of toxicity and the incidence of secondary malignancies.

In preclinical models, PARP inhibitors showed synergistic activity with immune checkpoint inhibitors,^32^ which has led to several ongoing clinical trials combining these two promising new classes of cancer therapeutic agents (see e.g. clinical trial NCT02657889). Our results strongly suggest that this synergistic effect is not due to the induction of neoepitopes by PARP inhibitors, but rather some other regulatory effect of immune response relevant gene regulation.^33^

We found a significant increase of microhomology-mediated deletions in niraparib-treated *BRCA1*^*−/−*^ DT40 cells. This effect may be related to the HPRT mutagenesis seen in *BRCA2* mutant Capan-1 cells.^12^ If PARP inhibition has a weak selective mutagenic effect on *BRCA1/2*-deficient cells only, this may accelerate the development of resistance in existing *BRCA*-deficient tumours, but would not contribute to the induction of secondary malignancies. We did observe an induction of SCEs as also reported for olaparib treatment,^14,15^ but this was not accompanied by mutagenesis. Apart from an increase in the number of large deletions in DLD-1 cells, we did not see an induction of chromosomal rearrangements, which might be an expected consequence of chromosome aberrations also reported for olaparib,^14^ but our assay here was of limited power for these rare events due to the low number of sequenced clones and the inability of the employed CREST algorithm to detect rearrangements with unmappable breakpoints.

A genetic loss of PARP-1 has been shown to accelerate the induction of mammary tumours in mice.^34^ PARP-1 deletion is not equivalent to inhibition by niraparib, which efficiently traps the enzyme on DNA,^10^ but further experiments would be helpful to test whether long-term in vivo PARP inhibitor treatment has a detectable carcinogenic effect.

The results also have a direct relevance to the evolution of resistance to PARP inhibitors. Two genetic mechanisms of emerging PARP inhibitor resistance in HR-deficient cells and tumours have been documented in the literature: suppressor mutations in genes such as *53BP1*^35^ and *REV7*,^36^ or secondary mutations that restore the function of the originally mutated HR genes such as *BRCA1*,^37^ *BRCA2*,^38,39^ *RAD51C* or *RAD51D*.^40^ We did not observe the evolution of resistance by either mechanism, though the large deletion in *BRCA1* in DT40 cells precluded genetic reversion. Importantly, mutagenic therapy can elicit such reversion mutations, both in tumours and in vitro within the 1-month timescale of cell culture model experiments.^16,38^ Our results suggest that unlike platinum agents, PARP inhibitor treatment will not induce mutations responsible for treatment resistance, and therefore the spontaneous mutagenic processes of the tumour and the choice of additional therapeutic agents will be most relevant to the rate of the evolution of PARP inhibitor resistance.

In conclusion, our comprehensive results revealed no mutagenic effect of niraparib apart from an increase in microhomology-mediated deletions in *BRCA1* mutant cells and an increase in large deletions in one cell type. While the long-term clinical relevance of such changes needs further study, our results suggest that niraparib treatment is unlikely to have more than a minor mutagenic effect on somatic and tumour cells.

## Electronic supplementary material

Supplementary Table 1

Supplementary methods

Supplementary figures

## Translational Relevance

PARP inhibitors are in use for the treatment of ovarian and breast cancer, and are undergoing trials on further cancer types. Due to their relatively mild side effects, PARP inhibitors have the potential for use in long-term maintenance treatment. However, inhibition of DNA repair with this family of drugs could potentially increase genomic mutagenesis, and the treatment-induced mutations could contribute to the development of resistance in the tumour or cause secondary malignancies in somatic cells. In this study, we used a combination of in vitro and in vivo approaches to show that long-term treatment with the PARP inhibitor niraparib has at most a minor mutagenic effect. In contrast to strongly mutagenic treatment with cisplatin, PARP inhibitors, therefore, represent a safer alternative for patients with good prognosis, and our results should increase the confidence in using PARP inhibitors for long-term treatments.
