# Supplementary material for: Long-term treatment with the PARP inhibitor niraparib does not increase the mutation load in cell line models and tumour xenografts
Source: Br J Cancer. 2018 Nov 14;119(11):1392–400. doi: 10.1038/s41416-018-0312-6 (PMC6265254; doi:10.1038/s41416-018-0312-6)
Supplement: Supplementary file 2 — Supplementary methods [file 41416_2018_312_MOESM2_ESM.docx]

**SUPPLEMENTARY METHODS**

*Sequence alignment*

Whole genome sequencing was done on Illumina HiSeq X Ten instruments using the 150 bp paired end format. The reads of DT40 cell lines were aligned to the chicken (Gallus gallus) reference sequence Galgal4.73 as described (1). Reads of human cell lines were aligned to the human reference GRCh38.p10. Duplicate reads were removed using samblaster (2). The aligned reads were realigned with GATK IndelRealigner (3). For the analysis of the patient derived xenograft data, the sequencing reads were first filtered for the presence of sequencing adapters by Trimmomatic (4) and aligned separately to the human reference sequence GRCh38.p10 and the mouse reference GRCm38.p5 by the Burrows-Wheeler Aligner (5). The reads better aligned to the human reference were selected using bamcmp (6).

*Copy number estimation in cell lines*

Copy number estimation for different regions along the genome was based on comparing sequenced reads with the reference genome. In a preliminary step, genomic positions with a reference allele frequency of 0.9 or larger were filtered out, while the reference allele frequency and a smoothed average coverage calculated in a short interval centred on the given position were recorded for the rest of the positions. From this data, the average depth ratio (determined from a raw estimate of the average diploid coverage) and the multiplicative inverse of the average reference allele frequency were combined to estimate copy number in consecutive regions using a running average method with a bin size of 1 Mb and an overlap of 50 kb. Whenever the two types of copy number measures suggested different actual copy numbers, the possibility of loss of heterozygosity was considered. The final copy number estimate for a given position was determined as the average copy number of multiple overlapping regions containing the position.

*IsoMut mutation filtering*

Independently arising SNVs and short indels were identified using the IsoMut tool developed for multiple isogenic samples (7). On DT40 and DLD-1 samples, IsoMut was run with default settings. Briefly, after applying a base quality filter of 30, data from all samples of one cell line were compared at each genomic position, and filtered using default parameters of minimum mutated allele frequency (0.2), minimum coverage of the mutated sample (5), and minimum reference allele frequency of all the other samples (0.93), and also filtered using the probability-based S quality score calculated from the mutated sample and one other sample with the lowest reference allele frequency. The S score filter was adjusted separately for SNVs, insertions and deletions based on maximum allowable false positive (FP) values such that no more than five FP SNVs, one FP insertion or one FP deletion would be identified in the starting clone.

In order to account for regions of different copy numbers in the SUM149PT cell clones during mutation detection, IsoMut was slightly modified so that in regions of p copy number, the default value of the min_sample_freq parameter was multiplied by 2/p. This way the minimal threshold of the non-reference allele frequency in the investigated samples was determined by the local copy number. Additionally, the optimisation of the S(p) score value was carried out separately for regions of different copy numbers. Given the FP total number of tolerable false positive events in the whole genome of a control sample as described above, the total number of allowable FP events in regions of a given copy number was reduced according to the proportion of the genome possessing that copy number. With all S(p) values set, mutations in all samples were filtered with the appropriate threshold for the score value determined by the local copy number.

To look for subclonal mutations unique to each whole exome sequenced PDX sample, the IsoMut tool was used with a minimum mutated allele frequency of 0.01, minimum coverage of 10 and a minimum reference allele frequency of 0.97 in all other samples. The IsoMut output was further filtered for at least three supporting reads and for position predicted to be in exons.

To identify mutations that are shared between the PDX samples belonging to the same xenograft line, the IsoMut tool was run separately for each sample together with the six PDX samples of the other line. After filtering the same way as above, only those positions were considered common PDX-specific SNVs that were present in all six samples of one PDX, but were totally absent from the other one.

*Identification of structural variations*

Structural variations were detected using the CREST algorithm (8) with a post-filtering step that required each breakpoint to be supported by at least five soft clipped reads. Additional filters were employed that removed all predicted structural variation breakpoints that were not unique among the samples or were in the 5 bps range of any exon boundaries.

*Generation and comparison of triplet SNV signatures*

96-triplet signatures (9) were generated after pooling samples of the same cell type and treatment. The mutation counts of each triplet categories were normalised accordingly with the actual triplet frequencies of the chicken or human genome. DT40 triplet signatures were adjusted with the ratio of each triplet occurrence in the human and chicken genome before comparison to the 30 human cancer triplet signatures (10) using Pearson correlation coefficient (11). For the visualisation of the relationships between out triplet mutation patterns and the COSMIC signature set, we used t-distributed stochastic neighbour embedding (t-SNE) with a perplexity parameter of 11.

**References for the supplementary methods**

1. Molnár J, Póti A, Pipek O, Krzystanek M, Kanu N, Swanton C*, et al.* The Genome of the Chicken DT40 Bursal Lymphoma Cell Line. G3 (Bethesda) **2014**;4(11):2231-40.

2. Faust GG, Hall IM. SAMBLASTER: fast duplicate marking and structural variant read extraction. Bioinformatics **2014**;30(17):2503-5.

3. McKenna A, Hanna M, Banks E, Sivachenko A, Cibulskis K, Kernytsky A*, et al.* The Genome Analysis Toolkit: a MapReduce framework for analyzing next-generation DNA sequencing data. Genome Res **2010**;20(9):1297-303.

4. Bolger AM, Lohse M, Usadel B. Trimmomatic: a flexible trimmer for Illumina sequence data. Bioinformatics **2014**;3(15): 2114-20.

5. Li H, Durbin R. Fast and accurate short read alignment with Burrows-Wheeler transform. Bioinformatics **2009**;25(14):1754-60.

6. Khandelwal G, Girotti MR, Smowton C, Taylor S, Wirth C, Dynowski M*, et al.* Next-Generation Sequencing Analysis and Algorithms for PDX and CDX Models. Mol Cancer Res **2017**;15(8):1012-6.

7. Pipek O, Ribli D, Molnar J, Poti A, Krzystanek M, Bodor A*, et al.* Fast and accurate mutation detection in whole genome sequences of multiple isogenic samples with IsoMut. BMC Bioinformatics **2017**;18(1):73.

8. Wang J, Mullighan CG, Easton J, Roberts S, Heatley SL, Ma J*, et al.* CREST maps somatic structural variation in cancer genomes with base-pair resolution. Nat Methods **2011**;8(8):652-4.

9. Alexandrov LB, Nik-Zainal S, Wedge DC, Aparicio SA, Behjati S, Biankin AV*, et al.* Signatures of mutational processes in human cancer. Nature **2013**;500(7463):415-21.

10. COSMIC. 2016 COSMIC: Signatures of mutational processes in human cancer. http://cancer.sanger.ac.uk/cosmic/signatures Accessed on 5 January 2018.

11. Szikriszt B, Poti A, Pipek O, Krzystanek M, Kanu N, Molnar J*, et al.* A comprehensive survey of the mutagenic impact of common cancer cytotoxics. Genome Biol **2016**;17:99.
