# Supplementary material for: Long-term treatment with the PARP inhibitor niraparib does not increase the mutation load in cell line models and tumour xenografts
Source: Br J Cancer. 2018 Nov 14;119(11):1392–400. doi: 10.1038/s41416-018-0312-6 (PMC6265254; doi:10.1038/s41416-018-0312-6)
Supplement: Supplementary file 3 — Supplementary figures [file 41416_2018_312_MOESM3_ESM.pdf]

Figure S1

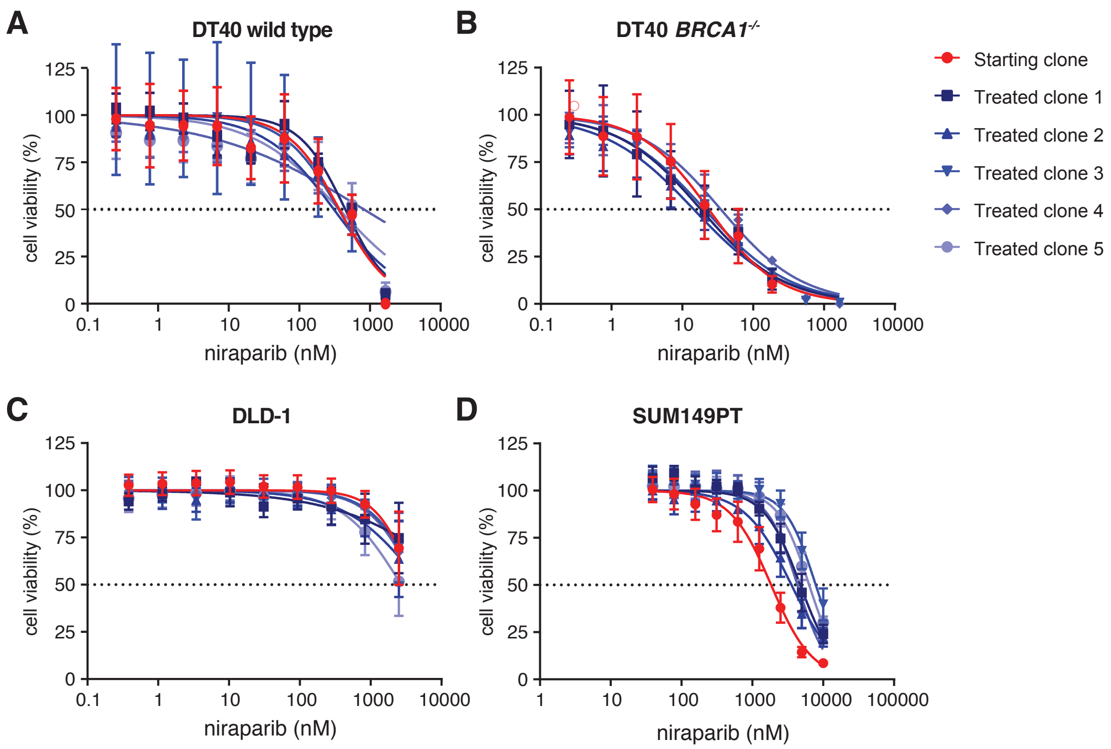

Figure S1. The effect of long-term niraparib treatment on niraparib sensitivity

(A-D) Comparison of the niraparib sensitivity of the starting clone and five post-treatment cell clones of each indicated cell line using cytotoxicity assays. The mean and S.E.M. of three independent experiments is shown throughout.

Figure S2

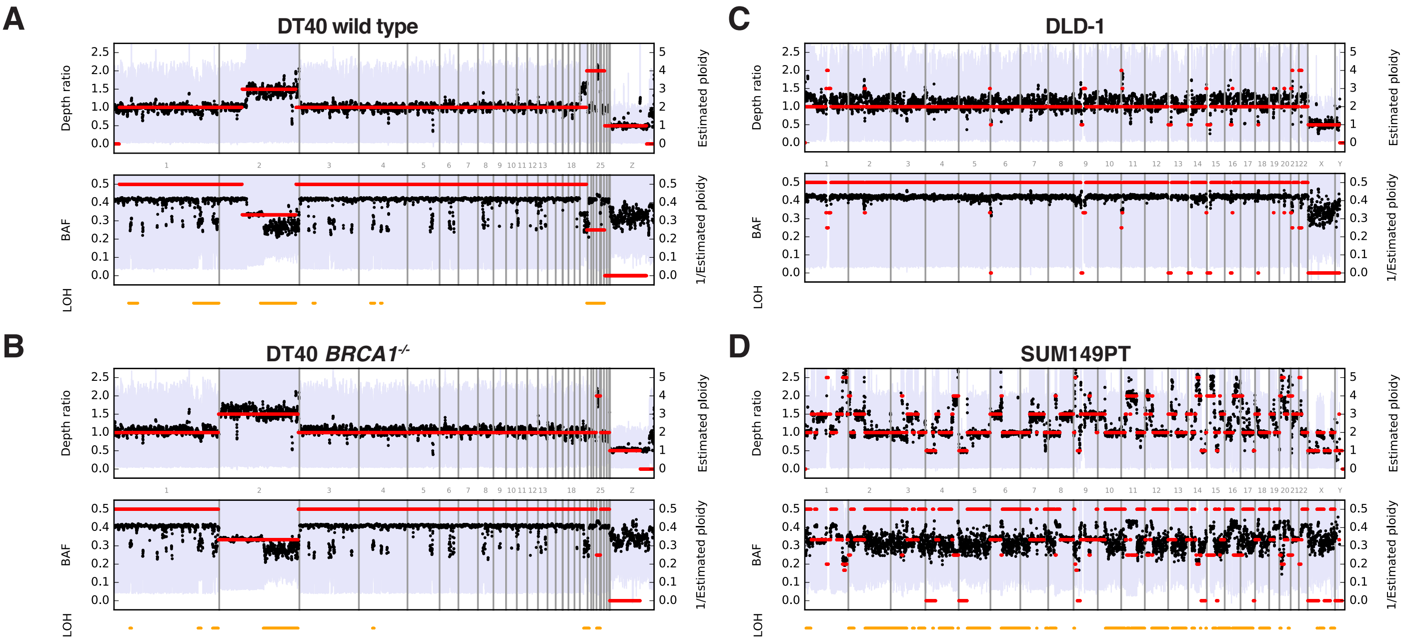

Figure S2. Karyotype analysis of the starting cell clones

(A-D) Copy number and heterozygosity information of each cell line derived from whole genome SNV data, displayed along the genome of the indicated cell line starting clone. Top panel, depth ratio derived from sequence coverage at non-reference SNVs compared to a diploid region; bottom panel, B allele frequency (BAF) of non-reference SNVs. Black dots show an average value for events in a local region of the genome with a bin size of 1 Mb and an overlap of 50 kb. Grey error bars represent the interquartile ratio for each calculated average value. A continuous red line shows the modelled ploidy (copy number) of chromosomal regions. Orange lines below the panels indicate regions of loss of heterozygosity (LOH). Chromosome boundaries are indicated with vertical black lines, chromosome numbers are shown between the panels.

Figure S3

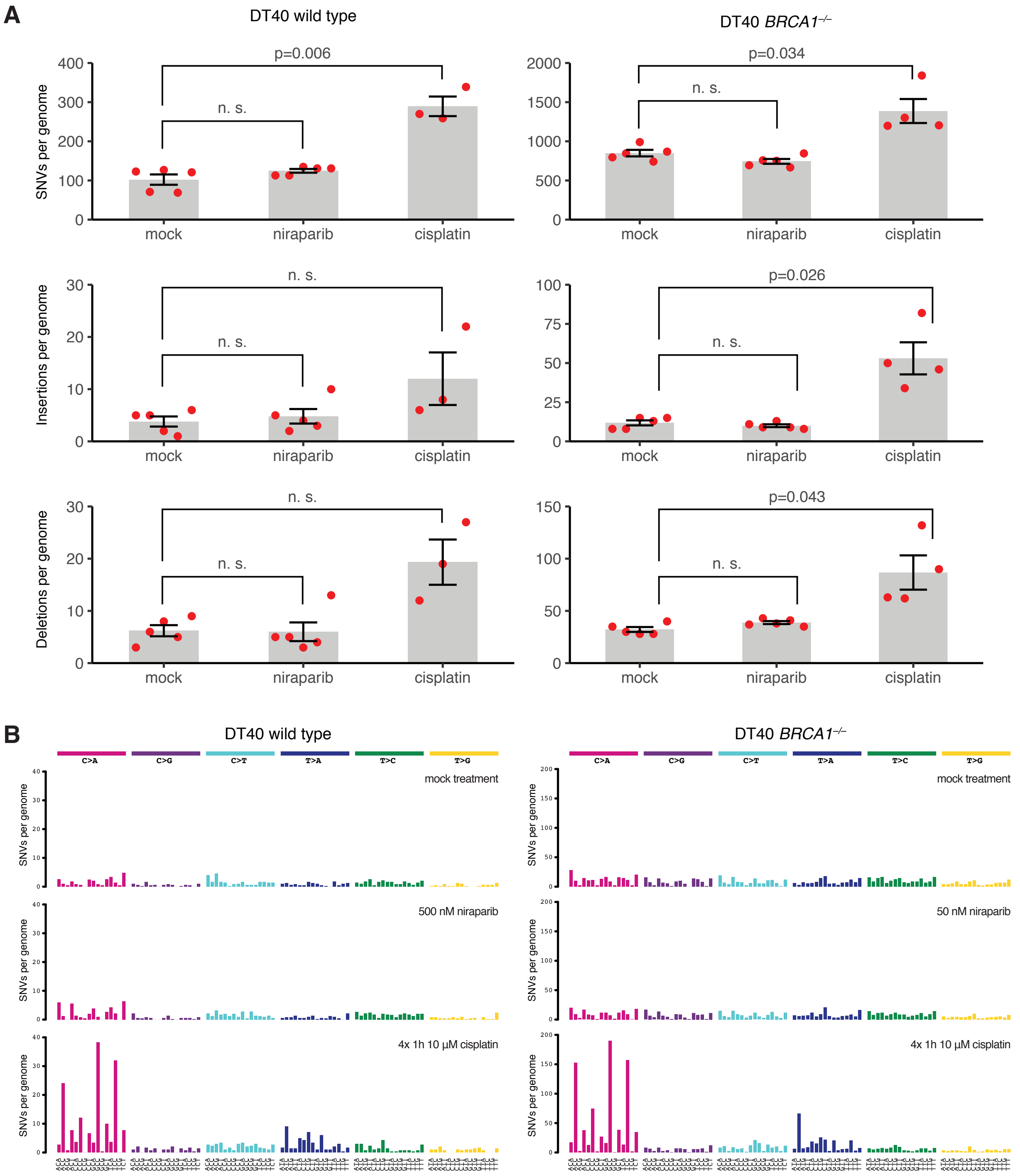

Figure S3. A comparison of the mutagenic effect of treatment with niraparib or cisplatin. 30-day niraparib treatment is compared to four weekly cycles of 1 hour cisplatin treatment as described in the materials and methods section. The overall duration of the treatment regimens was identical. (A) The mean number of SNVs, short insertions or short deletions in wild type DT40 cells (left panels) or *BRCA1*$^{-/-}$ mutant DT40 cells (right panels). Red symbols show the values for individual samples, error bars indicate S.E.M. The p values of significant changes are shown (*t*-test). (B) Mean triplet SNV mutation spectrum of the mock, niraparib or cisplatin treatment in the indicated cell lines. Each mutation class, as indicated at the top of the panel, is separated into 16 categories based on the identity of the preceding and following nucleotide as shown below.

Figure S4

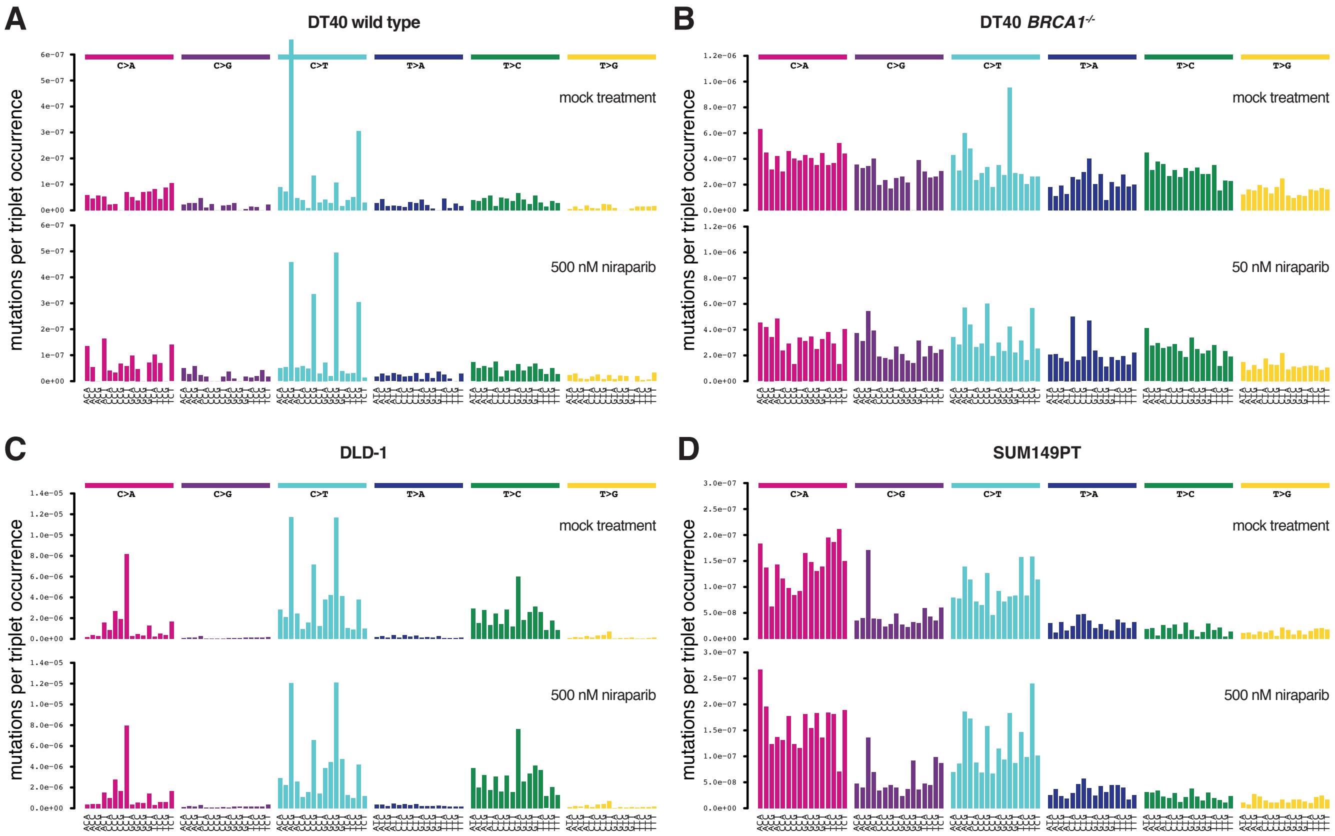

Figure S4. SNVs generated during long-term niraparib treatment

(A-D) Mean triplet SNV mutation spectrum of the mock treatment (top panel) or niraparib treatment (bottom panel) in the indicated cell lines. Each mutation class, as indicated at the top of the panel, is separated into 16 categories based on the identity of the preceding and following nucleotide as shown below. The mutation rate at each triplet was normalised to the number of occurrences of the triplets in the chicken or human genome.

Figure S5

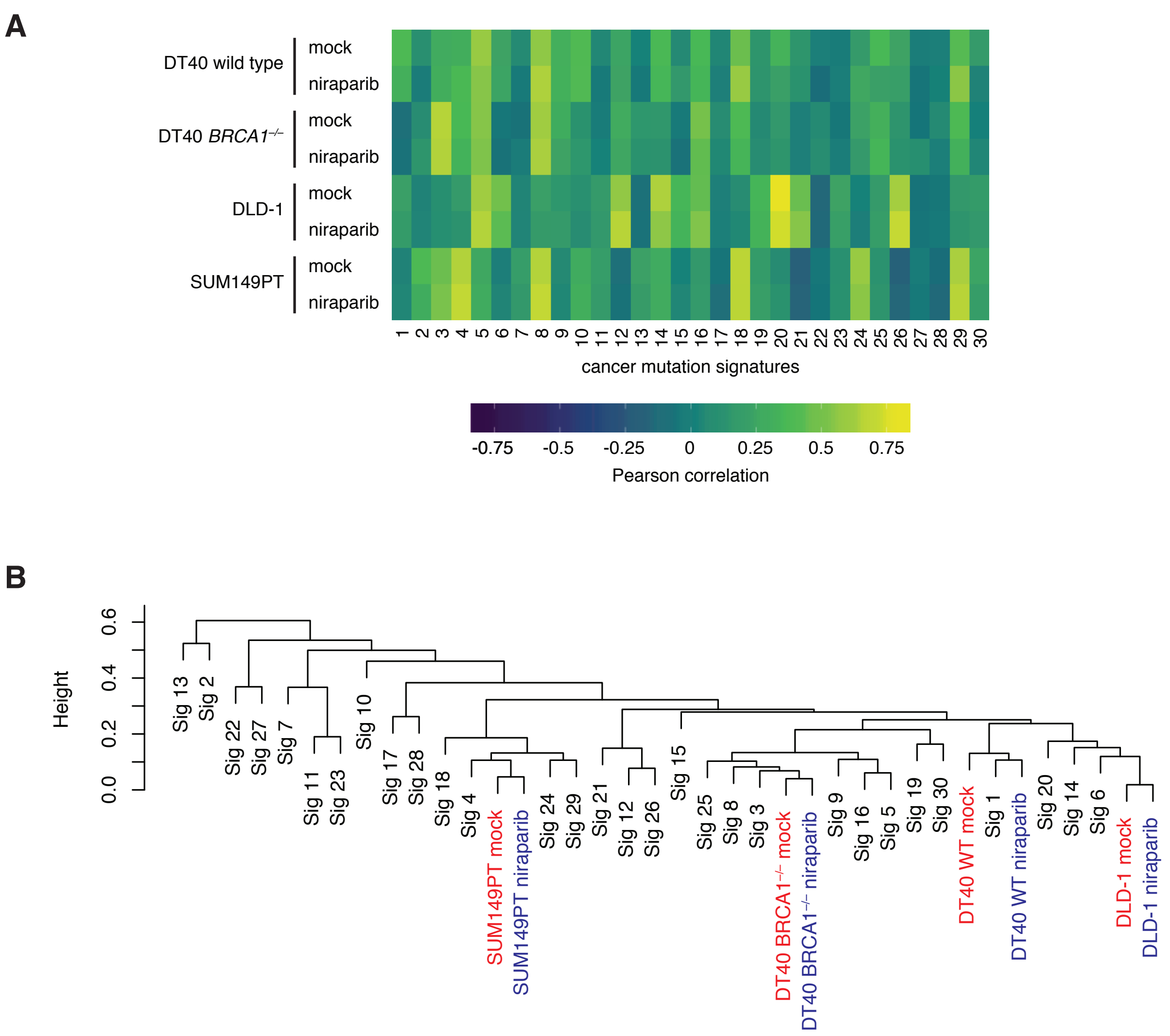

Figure S5. Correlation of triplet SNV spectra detected in cell culture experiments with mutational signatures identified in cancer

(A) Heat map of the Pearson correlation coefficient between mean triplet base mutation patterns observed in each indicated cell type and treatment, and the 30 COSMIC mutational signatures identified in human cancer. The heat map keys are shown below the panel. (B) Hierarchical clustering of the same triplet patterns and COSMIC signatures.
